# The Role of Family Influence and Academic Satisfaction on Career Decision-Making Self-Efficacy and Happiness

**DOI:** 10.3390/ijerph18115919

**Published:** 2021-05-31

**Authors:** Orhan Koçak, Namık Ak, Sezer Seçkin Erdem, Mehmet Sinan, Mustafa Z. Younis, Abdullah Erdoğan

**Affiliations:** 1 Faculty of Health Science, Istanbul University—Cerrahpasa Istanbul, 34320 Istanbul, Turkey; abdulllaherdogan@gmail.com; 2 Faculty of Engineering, Karamanoğlu Mehmetbey University, 70110 Karaman, Turkey; namikak@kmu.edu.tr; 3 Vocational School of Technical Sciences, Istanbul University—Cerrahpasa Istanbul, 34320 Istanbul, Turkey; erdem@iuc.edu.tr (S.S.E.); mehmet.sinan@iuc.edu.tr (M.S.); 4 College of Health Sciences, Jackson State University, Jackson, MS 39217, USA; younis99@gmail.com

**Keywords:** career decision, family influence, self-efficacy, happiness

## Abstract

Careers are a reality of life that need to be considered as multi-dimensional in today’s modern societies. Choosing a career is a complex process that coincides with high school and university ages, creating psycho-social stress. Considering the literature, the effects of different environmental factors have been revealed in separate studies. This study examines both individual and environmental factors together. By adopting a quantitative research method, we collected cross-sectional data through online questionnaires from 1130 university students. The association of family influence and academic satisfaction with happiness through career decision self-efficacy was meaningful using gender, age, income, and parents’ education as control variables. Family influence and academic satisfaction were positively correlated with career decision self-efficacy and happiness. In conclusion, we found that family influence and support, students’ work, and academic satisfaction are positively significant in terms of the career process and happiness. It was understood that the career reality should be considered with a holistic view that includes family, school, and work experience.

## 1. Introduction

Career expectations have become an essential concept in professional life and have been defined by many researchers with different perspectives. Among these definitions, different definitions range from career being the sum of work, family, and school processes to being a lifestyle [1,2,3,4]. In addition, a common definition is that a career is a process that continues during an individual’s developmental period and throughout life [5,6,7]. Based on these definitions, a career is a component of all processes before, during, and after the choice of profession, including making an effort to be successful, and succeeding during these periods using various resources.

The turning point for choosing a career usually starts during the pre-university or university era. In this period, when individuals experience difficulties in both their psychological and social lives, asking them to make decisions that will affect their entire lives will increase individuals’ stress and prevent them from making sound decisions [6]. During the high school years, individuals who do not yet fully know themselves and cannot identify their advantages, shortcomings, opportunities, and the dangers that their choices will cause may have to choose a profession and career that is not suitable for them later on. This will negatively affect the happiness levels of individuals and reduce their life satisfaction [8,9,10]. Individuals will consider some factors which have the power to affect them both positively and negatively, such as peer environment, guidance services, school experiences, and their family.

In this study, we focused on university students because university is a turning point for career decision making in Turkey. It was assumed that some demographic variables, family influence, and academic satisfaction would be associated with career decision self-efficacy and happiness based on the ecological concept which was developed by Bronfenbrenner [11]. The ecological concept considers a holistic approach to family influence, academic success, work experiences, parents’ education, and income level in the career process and is a notable part of the study. How students’ work experiences affect their career processes and their possible contribution to future working lives and labor market knowledge will be discussed. We found a significant positive impact of family influence and academic satisfaction on career decision self-efficacy and happiness in accordance with the ecological concept. Additionally, a significant mediation impact of career decision self-efficacy in the relationship between family influence, academic satisfaction, and happiness was observed. A moderating impact of parents’ education between academic satisfaction and happiness, and also career decision self-efficacy and happiness, was found. In all analyses, gender, age, class level, income level, parents’ education level (mean), and working status of participants were used as control variables.

### 1.1. Career Decision-Making Self-Efficacy (CDSE)

The process called career decision making is a critical period that affects the future of individuals together with their families. Career choice will determine the individual’s quality of life. Remarkably, for people who spend most of their lives in their jobs, career choice is a factor that directly affects happiness [12,13].

Choosing a profession that an individual wants to do and making an effort to prepare for that profession is called the career process [14]. The most important factors in an individual’s decision are their strengths and weaknesses. When choosing a career path, individuals will decide on their preferences, considering the effects of their physical and mental abilities, their academic skills, and economic situation [15,16,17,18]. Even though it is sometimes beneficial for better career opportunities, changing career path after studying at university is both challenging and wasteful of resources [19,20,21]. Therefore, for their career choice, individuals should be supported and show compatibility between their characteristics and their needs and expectations [13,15].

Career decision making is one of the issues that needs to be emphasized when planning for the future [15]. Happiness and relationships with people will be directly affected by the possible problems of individuals who have problems related to their career decision [13]. It is difficult and complicated to make an important decision for life before university. Individuals should compare their social, physical, and mental characteristics with their chosen profession’s features to facilitate the career decision process and examine possible problems in advance that may arise later [22]. Therefore, self-efficacy is crucial in making career decisions. Career decision self-efficacy is defined as the degree of belief that individuals can perform the career process successfully [23].

Many factors, such as friendships, expectations from a profession and employment, societal perception, academic satisfaction, personal characteristics of the individual, and their family’s influence, affect the career decision process [24,25,26,27]. These systems have complex relationships with each other, and they can affect each other. While the studies conducted in this field mainly focus on the individual factors, namely their abilities, values, and interests, environmental factors, especially the family and education, are also keystones of this process [28,29].

### 1.2. Family Influence and Academic Satisfaction on Career Decision Self-Efficacy

Our study was based on the ecological concept in which family influence and income, parents’ education, participation in working life, and academic satisfaction are essential elements [11,28,30]. The ecological concept has four basic systems, which are micro, mezzo, eco, and macro, which affect the individual in the career choice process [31]. The microsystem is individual, the mezzosystem is family and peers, the ecosystem is relatives and neighbors, and the macrosystem is ideological groups [30]. Additionally, another theory is used in the literature called the social cognitive theory of careers developed by Lent et al. [32], which focuses on academic success and family support [33,34]. Additionally, many other theories include family influence and academic output in their models [20,25,35,36,37].

Each family and culture follows a development process of its own, and therefore there will be differences among individuals [36,38,39]. The existence of these traditional, cultural, and social differences is an advantage for societies. However, these differences are expected to positively affect young people’s career decisions and happiness [6]. In the literature, family support is associated with many variables. It was determined that family influence significantly affects professional improvement, and as the support felt by young people increases, their professional improvement increases in parallel [34,40,41,42]. Additionally, a negative relationship was found between the social support level of the family and professional indecision since individuals are preoccupied by their families’ recommendations [43,44,45,46]. It was found that family support is a significant factor in the expectation of professional outcomes [47,48]. Studies show that the career decision-making process is positively affected by an increase in the level of social support perceived from parents [49,50]. Family support has a significant effect on overcoming occupational barriers as well as gender, ethnicity, and socio-economic status [51,52]. The influence of the family on the child is an undeniable fact. From the child’s development to character formation and career processes, the family is influential [53,54,55]. In light of the literature, we can consider that family influence is very effective on CDSE since the family transfer their knowledge and experiences, give financial support, teach their societal values, and give help during tough times in terms of career and other issues to their children.

**Hypothesis** **1** **(H1).** *Family influence has a positive effect on career decision-making self-efficacy.*

Research shows that individuals’ career decision-making self-efficacy is mostly influenced by their families and academic satisfactions. Families are very influential in shaping their children’s interests and values, developing self-concepts, and giving positive and negative perspectives on professions [1,35,56]. In addition, academic satisfaction is another factor that impacts career decision-making self-efficacy, and experiences, skills, and competencies learned in school can preferably be turned into career decision self-efficacy. Career decisions of university students will be affected by the following experiences at school: The quality of the education they receive from the school, whether the education they receive meets their needs when they graduate, their perceptions about how the education at the school will affect their career, their satisfaction with the department and their sense of belonging, whether they have any hopes to find a job after graduation, and how satisfied they are with the applied vocational education they received from the school [27,34,57,58,59].

**Hypothesis** **2** **(H2).** *Academic satisfaction has a positive effect on career decision-making self-efficacy.*

Baumrind et al. (2010) divided parental attitudes into democratic, authoritarian, and highly permissive. Democratic parents cause positive changes in high school adolescents’ career decisions [60,61]. According to Bi et al. (2018) [62], parental attitudes are divided into demandingness and responsiveness. With demands contrary to children’s self-perception, children are expected to do tasks that they cannot do or have difficulty doing. With responsiveness, the expectations of parents of their children are in line with their level and understanding. In this way, parents know and trust their children and believe they can do the task [63,64]. The attitudes of parents are related to their education level. Education levels of parents are effective in guiding children in every subject and making career decisions. In some countries in Europe, it was found that parents’ education also increases the education and career decision self-efficacy levels of their children [65,66]. Parents’ education affects their knowledge of the profession and life, their experiences, their horizons, and ultimately their decisions for themselves and their children. It is seen that well-educated families make an effort to prepare a better future for their children by taking advantage of this knowledge and experience [53,67,68,69]. Therefore, the influence of the family is expected to affect the child’s CDSE.

**Hypothesis** **3** **(H3).** *Parents’ education has a positive effect on career decision-making self-efficacy.*

### 1.3. Family Influence and Academic Satisfaction on Career Decision Self-Efficacy and Happiness

Happiness has evolved into a multifaceted idea that has captured humanity’s interest, especially in psychology and philosophical sciences. Happiness has been psychologically divided into four essential dimensions. There is happiness based on life satisfaction, happiness based on common sense, happiness based on perceived desire satisfaction, and happiness based on enjoyment [70]. The important thing here is the effect of working life, namely career choice, on happiness [71,72].

Career selection is a dynamic process affected by a variety of value judgments, desires, and beliefs. Individuals select their careers based on factors such as their preferences in the profession, morals, and the level of satisfaction they would get, as well as their personal characteristics [73,74]. If individuals work in a suitable profession, it will inevitably provide physiological and psychological satisfaction [13]. However, if individuals have to work in an unsuitable profession due to not making the right decisions during the career choice process, psychological problems may arise in individuals and professional failure and inefficiency. Thus, the happiness levels of individuals will decrease in life. Additionally, modern life is shaped by the influence of family, friends, and career. However, family is the most fundamental factor among them and affects the others as well. In this sense, the positive effect of the family will allow children to make better decisions in terms of school, friends, and career processes and increase their happiness [75,76,77,78,79].

**Hypothesis** **4** **(H4).** *Family influence has a positive effect on happiness*.

Reaching happiness is meaningful and valuable in most societies. It was observed that many individuals regard happiness as the primary goal, and that is why most people seek happiness [80,81]. Many branches of science have studied happiness and determined that happiness is affected by many factors, such as geographical location, social support, career, health, gender, and education. In a study by Dean and Gibbs [82], academically satisfied students were more optimistic about their careers and future, as academic satisfaction ensures that students’ positions in the labor market are guaranteed in some way. Furthermore, students with academic satisfaction will have a better place in society and a better chances in employment, increasing their happiness [83,84,85,86,87,88,89].

**Hypothesis** **5** **(H5).** *Academic satisfaction has a positive effect on happiness.*

Education, in every way, is the most critical investment in people’s futures. Although individuals need to have a good education, their parents’ education also contributes to the future of both themselves and their children [90,91,92,93], as educated parents will try to make a positive contribution to their children’s lives in general and their education and professional life in particular. They will use their knowledge, experience, opportunities, and financial resources to enable their children to have a better future, job, career, and opportunities. Some parents have high expectations while supporting their children, causing some children to be more stressed and unhappy, while some are happier [94,95,96].

**Hypothesis** **6** **(H6).** *Parents’ education has a positive effect on happiness.*

In the modern period, life revolves around careers. That is why individuals’ happiness is closely related to their careers. When examined from an individual perspective, factors affecting happiness are family and friends, income, working life, and education status in the modern world [97,98,99,100,101]. Therefore, happiness is affected by career choice and profession, and career choice is impacted by family influence, academic satisfaction, and parents’ education and income level. According to the literature, it is seen that family influence, academic satisfaction, parents’ education, family income, and students’ work experiences affect career decision self-efficacy (CDSE), and CDSE affects students’ happiness [65,66,102,103,104]. Therefore, there is a mediating effect of CDSE in the relationship between dependent variables and happiness. According to the literature, the following hypotheses were determined.

**Hypothesis** **7** **(H7).** *There is a positive mediating effect between family influence and happiness through career decision-making self-efficacy.*

**Hypothesis** **8** **(H8).** *There is a positive mediating effect between academic satisfaction and happiness through career decision-making self-efficacy.*

**Hypothesis** **9** **(H9).** *There is a positive mediating effect between parents’ education and happiness through career decision-making self-efficacy.*

**Hypothesis** **10** **(H10).** *There is a positive mediating effect between income and happiness through career decision-making self-efficacy.*

**Hypothesis** **11** **(H11).** *There is a positive mediating effect between type of work contract and happiness through career decision-making self-efficacy.*

The intellectual capacity of parents has an essential place in the development process of children [105,106]. As explained above, when parents have a higher education level, this positively affects the academic and social development of children [90,91,92,93,107,108]. In this sense, there may be a moderation effect as the parents’ education level can be significant leverage in their children’s future. That is why we thought that parents’ education levels might have a moderating effect on the impact of the students’ family influence, the career decision-making competence of the students, and on the effect of academic satisfaction levels of the students on the happiness of students, as educated families are more knowledgeable, experienced, and competent in terms of life, education, professions, and career paths. In some cases, the high education level of the parents causes high expectations of the children. This situation shows a decrease in the happiness of some children who cannot meet these high expectations [41,95].

**Hypothesis** **12** **(H12).** *There is a moderating effect of parents’ education between CDSE and happiness.*

**Hypothesis** **13** **(H13).** *There is a moderating effect of parents’ education between FIS and happiness.*

**Hypothesis** **14** **(H14).** *There is a moderating effect of parents’ education between academic satisfaction and happiness.*

**Figure 1:**
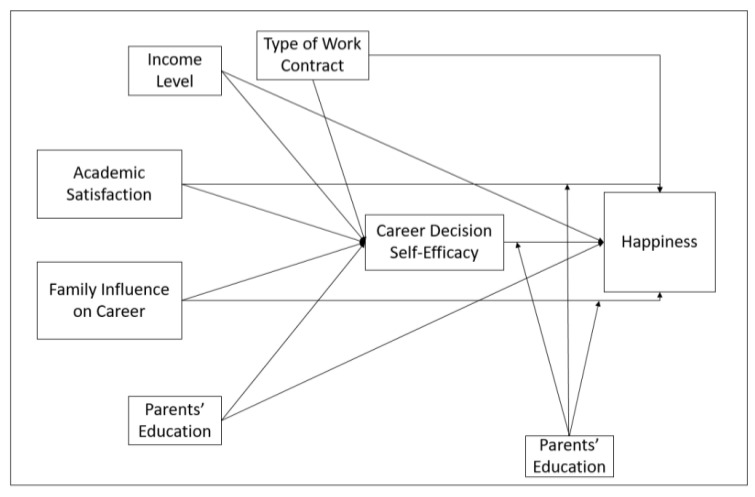
Research conceptual model.

To test these hypotheses, direct analyses were made between family influence, academic satisfaction, and career decision-making self-efficacy and happiness scales at the first stage. Afterward, direct effects were analyzed between five independent variables and the career decision-making self-efficacy and happiness. Finally, it was examined whether career decision-making self-efficacy has a mediating role between family influence, academic satisfaction, income level, type of work contract, parents’ education, and happiness, as shown in Figure 1.

## 2. Method

### 2.1. Study Design, Participants, and Procedure

We aimed to examine the effect of family influence, academic satisfaction, family income, parents’ education, and the type of work contract on the career decision-making self-efficacy and happiness of university students. When the studies in the literature were examined, we noticed that there is a need for studies related to environmental factors. Examining the family influence on academic satisfaction, which are the most critical environmental factors that affect an individual’s career decision self-efficacy, and also the impact on the happiness of individuals within the scope of the study, is essential.

The study design was cross-sectional, and a convenience sampling procedure was employed [109]. In order to detect if a common factor biased the results, we used Harman’s single factor test, and the score was less than 50% [110]. Therefore, common method bias does not affect the data and results. This study aimed to analyze the findings after establishing the relationship and impact rather than generalizing them.

The field study was carried out in different departments of universities in Istanbul using an online questionnaire. In the research group of the study, 1130 university students were determined by a simple random sampling method and participated voluntarily. Between 15 January and 25 February 2020, the survey was performed. Before completing the questionnaire, participants were briefed about the study’s methodology and objectives. Additionally, participants’ consent was taken prior to being asked to answer. The participants’ identifying details remained unknown since they were not requested. A necessary technical arrangement was made to ensure that the questions were answered only once. Participants were free to respond whenever they wanted. We maintained the data’s confidentiality and privacy. The research was carried out adhering to the Helsinki Declaration guidelines.

### 2.2. Data Analyses

After data collection through an online survey program, they were exported to MS Excel for cleaning and then imported into IBM SPSS 25 (IBM, Armonk, NY, USA). For demographics, the frequencies, averages, and standard deviations were determined by descriptive methods. After the factor analysis of all measures to ensure construct validity, correlations and regressions were performed. To perform multiple regression analysis, family influence, academic satisfaction, family income, parents’ education, and the type of work contract were defined as independent variables, happiness as the dependent variable, and career decision-making self-efficacy as a mediator variable in accordance with Figure 1. SPSS 22 for direct analysis and PROCESS Macro for SPSS (Model 4 and 15) software [111] for mediation and moderation analysis were used. To graph moderation effects and two-way interactions, a simple slope test was performed. The level of statistical significance was set at α > 95%.

### 2.3. Measures

#### 2.3.1. Sociodemographic Characteristics

Sociodemographic characteristics were asked for in the personal information form included in the study’s questionnaire. There were questions about university students’ gender, age, school types, departments, grade levels, education levels of their mothers and fathers, working status of their mothers and fathers, perception of socio-economic status, profession, and employment perceptions for their future. The questions about parents’ education were averaged by computing a single variable. Additionally, two dichotomous variables were generated for the type of work contract question to understand different groups’ feelings. For this purpose, full-time working students (coded 1) and others (coded 0), and part-time working students (coded 1) and others (coded 0) were re-evaluated.

#### 2.3.2. Family Influence Scale

The family influence scale in career development is a measurement tool developed to measure the family’s impact on the career development of individuals and is based on information about individuals. It was developed by [112] and adapted to Turkish culture by Akın et al. (2012) [113]. The scale, which consists of 22 items, includes statements such as “My family expects my profession to be in line with family values/beliefs” and “It is difficult for my family to support my professional decisions financially”. The scale includes a six-point Likert-type scale ranging from (1) Never Disagree (6) to Agree Fully. The total score for the scale is calculated. The higher the score, the higher the family influence on career development. The one-dimensional Cronbach’s alpha coefficient of the present study was determined as 0.851.

#### 2.3.3. Academic Satisfaction

Academic satisfaction was measured with four items that reflect the importance of academic satisfaction from the university department. The questions included “Are you happy with your department?”, “Will your department support finding a job?”, “How will your department contribute to your career?”, and “How appropriate is the knowledge you received in the department?”. A 5-point Likert-type scale was used, and a higher score means higher academic satisfaction. In factor analysis, the KMO value was found to be 0.771, and factor loads were found to be between 0.644 and 0.836. The explained variance of the factor was 64.132%. We found a one-dimensional factor which described academic satisfaction. The one-dimensional Cronbach’s alpha coefficient of the present study was determined as 0.809.

#### 2.3.4. Career Decision-Making Self-Efficacy Scale

The career decision-making self-efficacy (CDSE) scale was developed by Ulaş and Yıldırım (2016) in Turkey and consists of 45 items. The scale has a five-point Likert-type rating [114]. The total score that can be obtained from the scale is between 45 and 225. A high score obtained from the scale shows that university students have high career decision-making self-efficacy, and therefore they consider themselves capable of making career decisions. The one-dimensional Cronbach’s alpha coefficient of the present study was determined as 0.974.

#### 2.3.5. Happiness Scale

The happiness scale was developed by Demirci and Ekşi [115], consists of 6 items and one dimension, and there are no reverse coded questions. As a result of the factor analysis conducted to evaluate the happiness scale’s construct validity, which is a 5-point Likert-type scale, it was found that the scale has a one-dimensional structure with an eigenvalue of 3.248 and consists of 6 items explaining 54.129% of the total variance. The factor loads of the items in the scale range between 0.59 and 0.78. The Cronbach’s alpha internal consistency coefficient of the scale was calculated as 0.83. The test–retest reliability coefficient obtained by re-applying the scale to 62 participants with a difference of three weeks was found to be 0.73. The one-dimensional Cronbach’s Alpha coefficient of the present study was determined as 0.901.

## 3. Results

### 3.1. Descriptive Analyses

As seen in Table 1, 61.8% of the participants were women, 38.2% were men, and their average age was 21.97. It was understood that 52.8% of the students’ mothers were elementary graduates, 17.7% were middle school graduates, 19% were high school graduates, 9.4% were university graduates, and 1.1% were master’s or Ph.D. graduates. Additionally, we found that 33.5% of the students’ fathers were elementary graduates, 21.2% middle school graduates, 25.3% high school graduates, 17.3% university graduates, and 2.7% master’s or Ph.D. graduates. The average income level of students’ families was 4822.22. Of the students, 20.8% had a part-time working contract, and 5.8% had a full-time contract, whereas 73.5% of participants had no working contract, as seen in Table 1.

### 3.2. Correlation Analysis

Table 2 shows the means, standard deviations, and correlations of the scales with each other. The parents’ education variable was found to be positively associated with family influence (*r* = 0.201, *p* < 0.01), academic satisfaction (*r* = 0.129, *p* < 0.01), and with career decision self-efficacy (*r* = 0. 074, *p* < 0.05). The family influence variable was positively related to academic satisfaction (*r* = 0.146, *p* < 0.01), career decision self-efficacy (*r* = 0.284, *p* < 0.01), and to happiness (*r* = 0.325, *p* < 0.01). The academic satisfaction variable was positively correlated with career decision self-efficacy (*r* = 0.437, *p* < 0.01), and with happiness (*r* = 0.292, *p* < 0.01). The career decision self-efficacy variable was positively associated with happiness (*r* = 0.385, *p* < 0.01), as shown in Table 2.

### 3.3. Regression Analysis

In order to test some of our hypotheses, multiple regression analysis was performed between dependent variables and independent variables in three different models. In Model 1, age (*R* = 0.02, *p* < 001), part-time working (*R* = 0.13, *p* < 01), full-time working (*R* = 0.37, *p* < 001), family influence (*R* = 0.19, *p* < 001), and academic satisfaction had positive effects (*R* = 0.32, *p* < 001) on career decision self-efficacy (CDSE). In Model 2, gender (*R* = 0.23, *p* < 001), age (*R* = 0.02, *p* < 05), part-time working (*R* = 0.15, *p* < 05), family influence (*R* = 0.30, *p* < 001), academic satisfaction (*R* = 0.26, *p* < 001), and the interaction variable had positive (FIS X Parents’ Edu.) effects on happiness. According to Model 3 in Table 3, the impact of gender was positive (*R* = 0.25, *p* < 001), income was positive (*R* = 0.09, *p* < 001), parents’ education was negative (*R* = −0.09, *p* < 001), family influence was positive (*R* = 0.24, *p* < 001), academic satisfaction was positive (*R* = 0.15, *p* < 001), CDSE was positive (*R* = 0.33, *p* < 001), and the interaction variable (CDSE X Parents’ Edu.) was negative (*R* = −0.05, *p* < 05) on happiness. According to these results, hypotheses H1, H2, H4, H5, H6 were accepted, whereas hypothesis H3 was rejected.

### 3.4. Mediation Analysis

In our model illustrated in Figure 1, the mediating effect of CDSE on the impact of independent variables such as family influence, family income, academic satisfaction, type of work contract, and parents’ education on happiness was assumed. According to this model, direct regression analyses were performed between the independent, mediator, and dependent variables shown in Table 3. The SPSS Process Macro plugin was used to detect the indirect effect. According to the indirect regression effects shown in Table 4, it was seen that family influence and academic satisfaction maintained their effect on the dependent variable happiness through CDSE as a mediator. CDSE had a partially positive and significant effect as a mediator on the effect of family influence on happiness (γ = 0,0595, SE = 0,0127, 95% GA (0,0367, 0,0864)). CDSE had a partially positive and significant effect as a mediator on the effect of academic satisfaction on happiness (γ = 0,1118, SE = 0,0166, 95% GA (0,0798, 0,1457)). Additionally, it was seen that part-time working and full-time working did not maintain their effect on the dependent variable happiness through CDSE as a mediator. CDSE had a fully positive and significant effect as a mediator on the effect of part-time working on happiness (γ = 0,0416, SE = 0,0159, 95% CI (0,0131, 0,0756)). CDSE had a fully positive and significant effect as a mediator on the effect of full-time working on happiness (γ = 0,1214, SE = 0,0351, 95% CI (0,0555, 0,1915)). According to these values, it was understood that H7 and H8 that we predicted in our study were partly confirmed. Hypotheses H9 and H10 were rejected, whereas H11 was fully accepted.

### 3.5. Moderation Analysis

We tested moderation analyses in our model, as shown in Figure 1. Accordingly, to test the moderation impact of parents’ education, an interaction variable was generated between parents’ education and related academic satisfaction, family influence, and career decision self-efficacy variables. As a result of regression analyses in Model 2 and Model 3 in Table 3, the interaction effect of family influence and parents’ education on happiness (FIS X Parents’ Edu.) was not significant (*B* = 0.02, *p* > 0.05). However, two different interaction variables, which consisted of academic satisfaction (ACSAT X Parents’ Edu.) and career decision self-efficacy (CDSE X Parents’ Edu.), were found to be significant on happiness (*B* = −0.06, *B* = −0.05, *p* < 0.05, respectively). According to these moderation results, H12 was rejected, whereas H13 and H14 were accepted.

**Figure 2:**
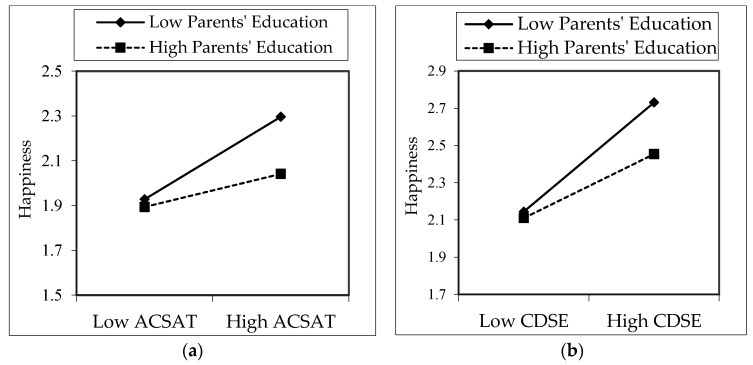
Moderation effect of parents’ education on the impact of ACSAT (**a**) and CDSE (**b**) on happiness.

The graphs of the moderation analysis are shown in Figure 2. Figure 2a shows that as the ACSAT levels of children of parents with high education levels rise, their happiness level falls lower than that of children of parents with low education levels. According to Figure 2b, it was seen that as the CDSE levels of the children of parents with high education levels increase, their happiness levels become lower than the happiness levels of the children of parents with low education levels.

**Figure 3:**
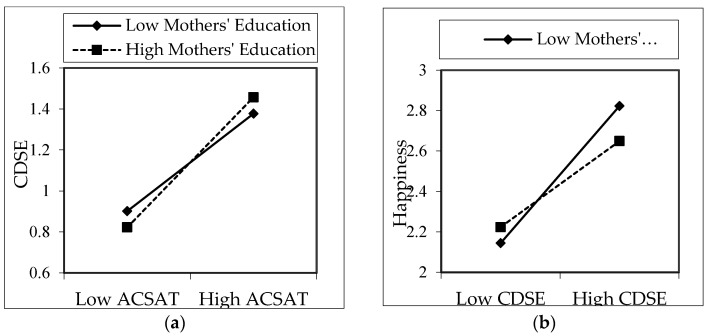
Moderation effect of mothers’ education on the impact of ACSAT (**a**) and CDSE (**b**) on happiness and CDSE (ad hoc analyses).

We conducted ad hoc analysis to see the moderation effects of the education levels of the mother and father separately. It was understood that only the education levels of the mothers had a statistically significant moderating impact. In Figure 3a, it is seen that as the ACSAT of those whose mothers have high education levels increases, their CDSE levels increase more than those whose mothers have low education levels (*B* = 0.094, *p* < 0.05). In addition, as shown in Figure 3b, it was seen that as the CDSE of those whose mothers have low education level increases, their happiness levels increase more than those whose mothers have a high education level (*B* = −0.188, *p* < 0.05).

## 4. Discussion

Career decision making is a required phase for individuals who are studying at a high school or a university. Career decisions will have an intense negative or positive effect until the end of individuals’ lives. However, the career process is also a burden for young people who are already under stress. That is why they need support from their social mechanisms such as family, school, and working life. In this study, we aimed to find the predictors of CDSE such as demographic variables, family influence, and academic satisfaction and, finally, their impact on the happiness of individuals. The study was based on the ecological concept, which was developed by Bronfenbrenner (1980). According to the results, hypotheses H1, H2, H4, H5, H6, H7, H8, H11, H13, and H14 were accepted, whereas H3, H9, H10, and H12 were rejected.

### 4.1. Effects on Career Decision Self-Efficacy

Family influence is one of the most crucial mechanisms during career decisions. We found a significant positive relationship between family influence and CDSE. Most of the literature considers family as a coping mechanism during career decisions, and family influence has a positive association with CDSE [30,33,42,116]. Parents’ attitudes towards essential decisions of their children enable children to perceive their support and overcome the challenges during the process. Trying to provide support without any coercion can contribute to children’s correct career decisions.

Another factor affecting CDSE is academic satisfaction, which was found to be positively significant in our study. Academic satisfaction is another aspect that influences CDSE, and experiences, abilities, and competencies acquired in school are ideally integrated into CDSE. The finding of academic satisfaction being associated with CDSE is consistent with the current literature [20,25,27,37,103,117]. Academic experiences are crucial not only for the career process and CDSE but also for the entire life course since they empower individuals to make decisions and cope with challenges.

We found that parents’ education has less than moderate (*r* = 0.074, *p* < 0.05) positive and significant correlation with CDSE, which is consistent with the literature [41,61,118]. Additionally, parents’ education was positively correlated with the academic satisfaction of their children. Therefore, we assumed that parents’ education level has a positive effect on CDSE, but the findings were not significant for the impact. However, when we carried out an ad hoc analysis about the moderation impact of mothers’ education on the association of academic satisfaction and CDSE, we found a significant positive effect. Accordingly, for those children who have mothers with high-level education, as academic satisfaction increases, their CDSE improves more than those who have mothers with low-level education. In a studies conducted by Pappas and Kounenou (2011) and Hsieh and Huang (2014) [119,120], similar results were found for both groups of mothers. In another ad hoc analysis, for the moderation of the impact of mothers’ education on the association of CDSE and happiness, we found a significant negative effect. Of children who have mothers with low-level education, as their CDSE increases, their happiness improves more than those who have mothers with high-level education.

### 4.2. Effects on Happiness

Many factors influence happiness, including geographical position, social support from family, occupation, health, gender, and education. Family influence was positively associated with happiness and had a positive impact on happiness. In the literature, there are similar results which show linkages between family influence and happiness [1,35,56]. Families have a large influence on their children’s preferences and beliefs, as well as on establishing self-concepts and providing positive and negative viewpoints on careers. Additionally, academic satisfaction was positively related to happiness which is consistent with the related literature [37,104,117,121]. Academic satisfaction has the power to directly affect happiness, as the contribution of academia to a person’s life will last for a lifetime.

Even though parents’ education was not associated with happiness, it negatively impacted happiness because of the other independent and control variables. Parents’ education and income are important parts of families’ socio-economic status, and both of them had a significant impact on happiness, which is consistent with the current literature [61,118,119,122]. In our study, the effect of parents’ education on happiness was negative, whereas income was positive. Highly educated parents may lead to higher expectations for and burdens on their children and thus decrease children’s happiness. However, children of high-income families may have more opportunities and thus increase their happiness.

### 4.3. Mediation Effects

We discovered partially significant mediating effects between family influence and happiness and between academic satisfaction and happiness through career decision-making self-efficacy. Through CDSE, the indirect effects of family influence and academic satisfaction on happiness were positive and meaningful. Nonetheless, there were no significant improvements in the direct outcomes of family influence and academic satisfaction on happiness. As a result, the mediating effect was statistically significant in part. We revealed that as the participants’ family support and influence increased during the career choice process, so did their self-efficacy in making career decisions and happiness. This outcome was found to be compatible with the literature [41,48,118]. According to studies, family influence and support benefit the job process and increase satisfaction from work and life. There were, however, no significant mediating effects between parents’ education and happiness, and between income and happiness, through career decision-making self-efficacy. Since there were no meaningful direct impacts of parents’ education and income on the mediator variable CDSE, indirect effects on happiness were not observed.

Through the CDSE of students, we discovered meaningful mediating impacts between part-time working, full-time working, and happiness. After examining the indirect relationship between part-time working, full-time working, and happiness through CDSE, the direct relationships between part-time working, full-time working, and happiness were found to be insignificant. As a result, the effects of CDSE’s mediating results between the type of work contract and happiness were fully significant. Accordingly, students working either part-time or full-time during their university years increase their CDSE levels and their happiness afterward. Studies found the working of students was associated with their CDSE [123,124,125]. Today, the most crucial problem for university students when finding a job after graduation is a lack of working experience. Therefore, the effect of students’ working will have a positive effect on both CDSE and happiness, which will help them to find employment after graduation.

### 4.4. Moderation Effects

We found moderation impacts of parents’ education between independent and dependent variables. According to the results, as the ACSAT levels of children of parents with high education levels rise, their happiness level falls lower than that of children of parents with low education levels. Additionally, as CDSE levels of the children of parents with high education levels increase, their happiness levels become lower than the happiness levels of the children of parents with low education levels. It was found that when the CDSE and ACSAT status of the children of families with low education levels increase, their happiness levels increase more than the other group. The children of families with a low level of education have to give more importance to their education and consequently their career processes; hence, their control in the process and, therefore, happiness are higher. However, we found a significant difference in the moderating of mothers’ education between ACSAT and CDSE. Thus, as highly educated mothers’ children’s ACSAT increased, the children’s CDSE improved more than mothers with a low level of education.

## 5. Limitations of the Study

Since the study was conducted only with the students of certain departments in universities in Istanbul and at a certain time, the results cannot be generalized to a different time or throughout the country. Additionally, conducting the study only online prevented observing the reactions of the participants. The study was conducted only with university students. However, doing similar studies with high school students and university graduates will make the career decision process more understandable. Another limitation is the cross-sectional design of the study and the fact that it was conducted only with students. For these reasons, the present results in the article cannot be generalized. Therefore, new research needs to be carried out with different groups and methods at other times.

## 6. Conclusions and Some Implications

With this study, we tried to find out the factors that affect the career decision self-efficacy of university students and, ultimately, their happiness. The study was conducted with 1130 students at different universities in Istanbul and was designed as cross-sectional, and a convenience sampling procedure was employed. As people are bio-psycho-social beings, both individual attributes and the social environment affect the career process. For this purpose, relationships with family income, family influence, parents’ education, academic satisfaction, and the type of work contract were examined in our study. It was found that family influence, academic satisfaction, parents’ education level, and working experiences significantly affected career decision self-efficacy and happiness. It was also found that career decision self-efficacy had a mediating effect and parents’ education had a moderating effect between dependent variables and happiness. We discovered that family influence and academic satisfaction positively impact students’ career decision self-efficacy and happiness. The most striking finding is that part-time and full-time students have higher career decision self-efficacies than non-working students and the full mediating effect of CDSE on the impact of their work experience on their happiness. However, there was no evidence of a mediating influence of CDSE on the correlation between family income, parental education, and happiness. 

For this reason, families should be informed and educated about support in children’s career processes. Considering the positive effect of family influence and support and the contribution of the education process on career decision self-efficacy, policymakers should do holistic planning that includes families, schools, neighborhoods, and children starting from primary school up to university. Training that will enable families to become more informed about their children’s career processes and support their children’s choices in accordance with their personalities should be provided. Especially during the university education of young people, policies should be strengthened to increase the application opportunities within universities and provide opportunities to work in the market in accordance with their education. Thus, they will combine theory and practice and have sufficient knowledge of the labor markets they can use after graduation. In this way, with an understanding that will put children and their future at the center, it will be ensured that children will learn and make more appropriate career decisions themselves.

## Figures and Tables

**Table 1:** Descriptive statistics.

		*f*	%	M	SD
Gender				1.62	0.49
	Female	698	61.8		
	Male	432	38.2		
Age				21.97	3.76
Mother’s Education				1.88	1.08
	Elementary	597	52.8		
	Middle School	200	17.7		
	High School	215	19		
	University	106	9.4		
	Master’s or Ph.D.	12	1.1		
Father’s Education				2.35	1.19
	Elementary	379	33.5		
	Middle School	239	21.2		
	High School	286	25.3		
	University	195	17.3		
	Master’s or Ph.D.	31	2.7		
Income Level				4882.22	5029.54
Type of Work Contract				1.32	0.58
	Not working	830	73.5		
	Part-time working	235	20.8		
	Full-time working	65	5.8		
Total		1130	100%		

**Table 2:** Means, standard deviations, and correlations.

No.	Variable	Mean	Std. Dev.	1	2	3	4
1	Parents’ Education (Mean)	2.11	1.00	1			
2	Family Influence	3.66	0.93	0.201 **	1		
3	Academic Satisfaction	3.68	0.87	0.129 **	0.146 **	1	
4	Career Decision Self-Efficacy	3.77	0.70	0.074 *	0.284 **	0.437 **	1
5	Happiness	3.75	0.93	0.022	0.325 **	0.292 **	0.385 **

** *p* < 0.01, * *p* < 0.05.

**Table 3:** Main effects on dependent variables.

Variable	Model 1: CDSE			Model 2: Happiness			Model 3: Happiness		
	B	SE	*p*	B	SE	P	B	SE	P
(Constant)	1.47	0.17	<0.001	0.81	0.23	<0.001	0.30	0.23	0.200
Gender 1 = m, 2 = f	−0.04	0.04	0.277	0.23	0.05	<0.001	0.25	0.05	<0.001
Age	0.02	0.01	0.000	0.02	0.01	0.024	0.01	0.01	0.212
Class	−0.01	0.02	0.554	0.03	0.02	0.275	0.03	0.02	0.175
Income	−0.02	0.02	0.196	0.09	0.03	0.001	0.09	0.02	<0.001
Part-Time Working	0.13	0.05	0.005	0.15	0.06	0.019	0.10	0.06	0.110
Full-Time Working	0.37	0.09	<0.001	0.20	0.12	0.103	0.07	0.12	0.532
Parents’ Edu. (mean)	0.01	0.02	0.628	−0.09	0.03	0.001	−0.09	0.03	<0.001
FIS	0.19	0.02	<0.001	0.30	0.03	<0.001	0.24	0.03	<0.001
Academic Sat.	0.32	0.02	<0.001	0.26	0.03	<0.001	0.15	0.03	<0.001
FIS X Parents’ Edu.				0.02	0.02	0.500			
ACSAT X Parents’ Edu.				−0.06	0.03	0.017			
CDSE							0.33	0.04	<0.001
CDSE X Parents’ Edu.							−0.05	0.02	0.045
F		50.74			27.66			35.07	
p		<0.001			<0.001			<0.001	
R^2^		0.290			0.214			0.257	

CDSE = Career Decision Self-Efficacy, FIS = Family Influence Scale, ACSAT = Academic Satisfaction.

**Table 4:** Total, direct, and indirect regression analysis on happiness.

				Unstandardized Effect		SE	LLCI	ULCI	
Total Effect of Family Influence on Happiness					0.2938	0.0277	0.2395	0.3480	Sig.
Direct Effect of Family Influence on Happiness					0.2343	0.0278	0.1798	0.2888	Sig.
Family Influence	>	CDSE	>	Happiness	0.0595	0.0127	0.0367	0.0864	Sig.
				Unstandardized Effect		SE	LLCI	ULCI	
Total Effect of Academic Satisfaction on Happiness					0.2655	0.0296	0.2075	0.3236	Sig.
Direct Effect of Academic Satisfaction on Happiness					0.1537	0.0317	0.0915	0.2159	Sig.
Academic Satisfaction	>	CDSE	>	Happiness	0.1118	0.0166	0.0798	0.1457	Sig.
				Unstandardized Effect		SE	LLCI	ULCI	
Total Effect of Part-Time Working on Happiness					0.1431	0.0631	0.0192	0.2670	Sig.
Direct Effect of Part-Time Working on Happiness					0.1016	0.0616	−0.0193	0.2224	N.S.
Part-Time Working	>	CDSE	>	Happiness	0.0416	0.0159	0.0131	0.0756	Sig.
				Unstandardized Effect		SE	LLCI	ULCI	
Total Effect of Full-Time Working on Happiness					0.2079	0.1213	−0.0302	0.4460	N.S.
Direct Effect of Full-Time Working on Happiness					0.0865	0.1189	−0.1468	0.3198	N.S.
Full-Time Working	>	CDSE	>	Happiness	0.1214	0.0351	0.0555	0.1915	Sig.

CDSE = Career Decision Self-Efficacy.

## Data Availability

The data presented in this study are available on request from the corresponding author. The data are not publicly available due to that they are a part of a developing dataset which will be used in the future for different studies.
